# Positive myositis-specific autoantibodies during COVID-19: a case report

**DOI:** 10.11604/pamj.2022.43.181.36322

**Published:** 2022-12-07

**Authors:** Asma Ben Abdelkrim, Mariam Ghozzi, Ghada Saad, Sarra Melayah, Taieb Ach, Ibtissem Ghedira, Koussay Ach

**Affiliations:** 1 Department of Endocrinology and Diabetology, University Hospital of Farhat Hached, Ibn Jazzar Avenue, 4031 Sousse, Tunisia,; 2 Department of Immunology, Farhat Hached Hospital University, Ibn Jazzar Avenue, 4031 Sousse, Tunisia,; 3 Research Laboratory for “Epidemiology and Immunogenetics of Viral Infections” (LR14SP02), Sahloul University Hospital, University of Sousse, Sousse, Tunisia,; 4 Faculty of Pharmacy, Department of Immunology, University of Monastir, Monastir, Tunisia,; 5 LR12SP11, Biochemistry Department, Sahloul University Hospital, Sousse, Tunisia

**Keywords:** COVID-19, myositis, autoimmunity, diabetes mellitus, case report

## Abstract

Viral infection is known to be a trigger of autoimmune diseases. Numerous cases of coronavirus disease 2019 (COVID-19) with autoimmune manifestations have been reported and several authors have highlighted the relationship between severe acute respiratory syndrome coronavirus 2 (SARS-CoV-2) and autoimmune diseases. Autoimmune myopathies being one of these manifestations. A 27-year-old diabetic woman was admitted for management of acido-ketosis decompensation of her diabetes secondary to SARS-CoV-2 infection. During hospitalization, she developed muscle weakness and increased creatine kinase levels, which led us to assay the autoimmunity pattern which became positive for myositis-specific autoantibodies against single recognition particle (anti-SRP). The patient was treated with intense hydration with clinical and biological improvement and anti-SRP disappeared two months later. Positive myositis auto-antibodies are one of the autoimmune complications that could be seen during and after the SARS-CoV-2 infection.

## Introduction

Coronavirus disease 2019 (COVID-19) is the first large-scale pandemic of the 21^st^ century. It is caused by a pathogen, the severe acute respiratory syndrome coronavirus 2 (SARS-CoV-2). COVID-19 first appeared in Wuhan, China in the fall of 2019, hence the name of COVID-19. The clinical presentation ranges from no symptoms to severe forms of life-threatening respiratory distress. The most reported symptoms are fever, fatigue and otorhinolaryngological manifestations including anosmia, ageusia, cough and nasal congestion. Gastrointestinal, ocular, cardiac and neurological manifestations have also been reported [1]. Viral infection is known to be trigger of autoimmune diseases. Numerous cases of the COVID-19 with autoimmune manifestations have been reported, and several authors highlighted the relationship between SARS-CoV-2 and autoimmune diseases [2]. Autoimmune myopathies being one of these manifestations. Several viruses have been implicated as possible triggers of myositis. With regard to the current COVID-19 pandemic, 10% of those who are infected with SARS-CoV-2 present with myopathic complaints of myalgia, weakness, and elevated creatine kinase (CK), sometimes to very high levels, reminiscent of necrotizing autoimmune myositis [3,4]. Here, we report a case of positive myositis auto-antibodies related to COVID-19 in a 27-year-old diabetic woman admitted for management of an acido-ketosis decompensation of her diabetes.

## Patient and observation

**Patient information:** a 27-year-old woman was admitted to the emergency room with abdominal pain and vomiting for three days. Cough, dyspnea and other respiratory symptoms were not observed. She was obese with type 2 diabetes mellitus (T2D) since 2012 on oral anti-diabetics.

**Clinical findings:** on admission, the patient was febrile at 40°C. She had a tachycardia at 100 beats per minute and a normal blood pressure at 11/7. She was eupneic at 18 cycles/minute with a peripheral oxygen saturation of 98%. The neurological and muscular examination was without abnormalities. Her weight was 77 kg with a height of 1.57 m (body mass index = 31.2 kg/m^2^). On day 4 of hospitalization the patient was apyretic, however, she presented muscle weakness and myalgia in the lower limbs that were disabling. The clinical examination showed a sensitivity of the lower limbs, with a decrease in muscle strength. Neurological and skin examinations were without abnormalities.

**Diagnostic assessment:** finger glucose was elevated to 4.2 g/l and urinalysis revealed acetoneuria. An arterial gasometry was performed revealing an uncompensated metabolic acidosis: pH 6.97, HCO_3_-: 4 mmol/L, PaCO_2_: 16 mmHg, PaO_2_: mmHg, with a functional renal failure in biology (creatinine: 203 μmol/L urea: 22 mmol/L). C reactive protein (CRP) was slightly increased at 12 mg/L and CK normal at 102 IU/L. The patient performed COVID-19 swab, by Real Time Reverse Transcription-Polymerase Chain Reaction (rRT-PCR), which was positive. On day 4, urgent laboratory examinations indicated an increase in CK to 3730 UI/L (normal range 38-234 UI/L) and elevation of CRP to 102 mg/L (normal range ≤ 10 mg/L), lactate deshydroganase (LDH) was 441 UI/L (normal range 135-250 UI/L), aspartate aminotransferase (AST) was 103 UI/L (normal range 5-40 UI/L), alanine aminotransferase (ALT) was 99 UI/L (normal range 5-40 UI/L). Complete blood count was without abnormalities. The immunological workup showed positive myositis-specific autoantibodies against single recognition particle (anti-SRP). Autoantibodies were determined using a multiplexed Euroline profile autoimmune myositis kit (EUROIMMUN®, Lübeck, Germany).

**Therapeutic intervention:** the patient was put under antibiotic therapy, anticoagulation and intravenous insulin therapy and hydration, the acid-ketotic decompensation disappeared in 3 days with the following results: pH 7.43, HCOPaO_3_-: 19.7 mmol/L, PaCO_2_: 27 mmHg, PaO_2_: mmHg, creatinine: 55 μmol/l, urea: 22 mmol/l. In addition to the previous treatments, the patient was immediately treated with hydration with gradual improvement in myalgia and muscle weakness. On day 7 of hospitalization, she was again able to move her lower limbs freely and the CK level dropped to 1155 UI/L.

**Follow-up and outcomes:** respiratory status was still stable, with a peripheral oxygen saturation of 98%. The patient´s symptoms improved daily, and biochemistry indicators gradually returned to normal levels (Table 1). Throughout her hospital admission, she didn´t present respiratory symptoms. The patient was discharged at day 13 of hospitalization on insulin therapy for her diabetes. Anti-SRP antibodies performed two months after her discharge were negative.

**Table 1:** clinical and biological characteristics in the follow-up

	Day1	Day2	Day3	Day4*	Day5	Day6	Day7	Day8	Day9	Day10	Day11	Day12
CK (38-234 UI/L)	102	ND	ND	3738	3200	2500	1155	558	451	334	234	211
LDH1 (35-250 UI/L)	ND	ND	ND	441	400	377	339	277	305	256	211	193
AST (5-40 UI/L)	ND	ND	ND	103	90	82	63	ND	38	32	24	22
ALT (5-40 UI/L)	ND	ND	ND	93	96	96	109	ND	82	61	50	40
CREAT (60 -120 µmol/l)	203	85	55	52	ND	ND	34	39	43	ND	ND	ND
CRP (≤ 10 mg/L)	36	ND	ND	102	ND	ND	12	ND	5	ND	ND	ND
pH (7.38-7.42)	6.97	7.26	7.40	7.43	7.42	ND	ND	ND	ND	ND	ND	ND

* Rhabdomyolysis symptoms appeared on day 4 CK: creatine kinase; AST: aspartate aminotransferase; ALT: alanine aminotransferase; LDH: lactate dehydrogenase; CREAT: creatinine; CRP: C reactive protein; ND: not done

**Informant consent:** written informed consent was obtained from the patient to publish anonymised information in this article.

**Diagnosis:** a ketoacidosis decompensation of type 2 diabetes secondary to SARS-CoV-2 infection complicated with autoimmune manifestations was confirmed.

## Discussion

We here report a case of a young woman admitted for ketoacidosis decompensation of her diabetes secondary to SARS-CoV-2 infection and who suffered during her infection from muscle pain and weakness. Anti-SRP antibodies were detected. SRP antibodies were found to be associated with high CK levels and can be detected in a subset of 4-6% of patients with myositis and almost 60% of patients with necrotic myositis [5]. Recent studies revealed the presence of auto-antibodies in patients with infection by SARS-CoV-2 and demonstrated that auto-antibodies are correlated with the severity of COVID-19 [6]. Our patient suffers from type 2 diabetes and obesity, which are well recognized determinants of increased severity related to acute COVID-19 [7]. In fact, the elevated glucose levels in human monocyte supports SARS-CoV-2 replication and proliferation [7]. During her infection, our patient suffers from myalgia. Despite that myalgia was described during COVID-19 in up to 50% of patients depending on the studies, myositis was only described in case reports [8]. In some cases, no myositis-specific auto-antibodies has been detected [8]. In our case, the patient had anti-SRP antibodies. Anti-SRP antibodies are associated with a low muscle strength score, a high CK levels and a severe degree of necrosis. Organ damage in COVID-19 was partly attributed to type I interferon, which is well known to play a major role in myofiber damage in myositis [9]. Several mechanisms could explain autoimmune manifestations during SARS-CoV-2 infection. In fact, humoral and cellular auto reactivity during or succeeding a viral infection is due to molecular mimicry between viral antigens and auto-antigens. SARS-CoV-2 had at least one cross-reacting epitope with human proteins [10]. Furthermore, in their study, Megremis S *et al*. [10] identified three immunogenic linear epitopes with a high sequence identity to SARS-CoV-2 proteins in autoimmune dermatomyositis. These cross-reacting epitopes lead to the loss of tolerance to self peptides. Bystander activation and damage is another mechanism which could explain autoimmunity in viral infection. In fact, it starts with the migration of cytotoxic CD8 T cells to the infection site and the release of perforin and granzyme. CD4 T cells intervene by exerting proinflammatory cytokines, enhancing phagocytic activities. The ineffective clearance of killed cells exposes auto antigens, resulting in auto-immunity [10].

## Conclusion

After almost three years of the emergence of this new coronavirus, several complications during and after the SARS-CoV-2 infection have been reported among which, the autoimmune complications take an important place. In our clinical case, we have reported one of these complications: positive myositis auto-antibodies related to COVID-19 which disappeared spontaneously after the acute phase of the infection.
